# Reproducibility and Bias in Healthy Brain Segmentation: Comparison of Two Popular Neuroimaging Platforms

**DOI:** 10.3389/fnins.2016.00503

**Published:** 2016-11-09

**Authors:** Dana L. Tudorascu, Helmet T. Karim, Jacob M. Maronge, Lea Alhilali, Saeed Fakhran, Howard J. Aizenstein, John Muschelli, Ciprian M. Crainiceanu

**Affiliations:** ^1^Department of Internal Medicine, University of PittsburghPittsburgh, PA, USA; ^2^Department of Biostatistics, University of PittsburghPittsburgh, PA, USA; ^3^Department of Psychiatry, University of PittsburghPittsburgh, PA, USA; ^4^Department of Biomedical Engineering, University of PittsburghPittsburgh, PA, USA; ^5^Biostatistics Program, Louisiana State University Health Sciences CenterNew Orleans, LA, USA; ^6^Department of Neuroradiology, Barrow Neurological InstitutePhoenix, AZ, USA; ^7^Department of Radiology, Banner Health and Hospital SystemsMesa, AZ, USA; ^8^Department of Biostatistics, Bloomberg School of Public Health, John Hopkins UniversityBaltimore, MD, USA

**Keywords:** MRI reproducibility, segmentation bias, healthy brain segmentation

## Abstract

We evaluated and compared the performance of two popular neuroimaging processing platforms: Statistical Parametric Mapping (SPM) and FMRIB Software Library (FSL). We focused on comparing brain segmentations using Kirby21, a magnetic resonance imaging (MRI) replication study with 21 subjects and two scans per subject conducted only a few hours apart. We tested within- and between-platform segmentation reliability both at the whole brain and in 10 regions of interest (ROIs). For a range of fixed probability thresholds we found no differences between-scans within-platform, but large differences between-platforms. We have also found very large differences between- and within-platforms when probability thresholds were changed. A randomized blinded reader study indicated that: (1) SPM and FSL performed well in terms of gray matter segmentation; (2) SPM and FSL performed poorly in terms of white matter segmentation; and (3) FSL slightly outperformed SPM in terms of CSF segmentation. We also found that tissue class probability thresholds can have profound effects on segmentation results. We conclude that the reproducibility of neuroimaging studies depends on the neuroimaging software-processing platform and tissue probability thresholds. Our results suggest that probability thresholds may not be comparable across platforms and consistency of results may be improved by estimating a probability threshold correspondence function between SPM and FSL.

## Introduction

Magnetic Resonance Imaging (MRI) is widely used in clinical practice and research. While MRI acquisition techniques have standard protocols within institutions across the world, the population level analysis of MRI obtained from heterogeneous sources is still under intense methodological development. The current state-of-the-art for pre-processing MRI data is to use standard software packages and develop research-group-specific processing pipelines. In practice, the choice of processing steps and associated parameters can substantially affect brain measurements and the conclusions of the study. We focus on studying the reproducibility and bias of brain MRI segmentation software. We consider two popular neuroimaging software platforms, Statistical Parametric Mapping (SPM, http://www.fil.ion.ucl.ac.uk/spm/software/spm12) and FMRIB Software Library (FSL, http://www.fmrib.ox.ac.uk/fsl/index.html), and compare results on the Kirby21 dataset (Landman et al., 2011). Kirby21 is a publicly available dataset containing scan-rescan imaging sessions on 21 healthy volunteers with no history of neurological disorders. Multiple imaging modalities were acquired on these volunteers including a three-dimensional, T1-weighted, gradient-echo sequence (MPRAGE), fluid attenuated inversion recovery (FLAIR), diffusion tensor imaging (DTI), resting state functional magnetic resonance imaging (fMRI), B0, and B1 field maps. For the purpose of this paper we use only the MPRAGE structural images.

Tsang et al. (2008) have investigated segmentation methods using SPM5 and FSL. They compared the performance of the methods on a phantom dataset as well as on 32 healthy volunteers and showed that SPM5 was more accurate than FSL in terms of gray matter (GM)/white matter (WM) segmentation. A similar investigation (Kazemi and Noorizadeh, 2014) was performed with newer versions of SPM and FSL using both a simulated and a real dataset. Kazemi and Noorizadeh's investigation found that SPM performed better in terms of accuracy of segmentation than FSL on both real and simulated data with varying level of noise and intensity inhomogeneity. In addition they also investigated Brainsuite, which performed worse in terms of accuracy than both, SPM and FSL. Klauschen et al. (2009) compared the segmentation performance of SPM, FSL, and FreeSurfer (Fischl, 2012). Specifically, they found that SPM had higher sensitivity and that SPM/FSL performed similarly in calculation of volumes, however in terms of gray matter FreeSurfer, SPM, and FSL performed differently (from best to worst). The results of Kazemi and Noorizadeh and Klauschen and colleagues support the result of Tsang and colleagues that SPM performs better in terms of segmentation. Eggert et al. (2012) compared reliability and accuracy of gray matter tissue segmentation using SPM, VBM8 (http://www.neuro.uni-jena.de/vbm/; Ashburner and Friston, 2000), FSL, and FreeSurfer. In addition to differences in gray matter mean segmented volumes between segmentation algorithms, Eggert and colleagues observed that the segmentation is highly sensitive to the skull-stripping technique applied.

Using manual segmentations of the gray matter, white matter, and CSF, Mendrik et al. compared three well-known neuroimaging methods (FSL, SPM, and FreeSurfer), as well as several other custom methods (Mendrik et al., 2015). They found that SPM, FSL, and FreeSurfer performed, in this order, from best to worst. The authors proposed that FreeSurfer's poor performance might have been due to the low resolution of the structural scan used. However, in gray and white matter FreeSurfer outperformed FSL (Mendrik et al., 2015). These results seem to further support previous findings (when comparing the common neuroimaging methods).

Our approach adds to the literature in at least three novel ways. First, we compare tissue segmentation at different probability thresholds and in each subject's native space (compared to a standard neuroanatomical space). Second, we characterize the scan/rescan reproducibility using a repeated measures model that includes a factor for scan. The scan factor plays a very important role since it can be used to test whether, on average, there is a statistically significantly difference between scan and rescan regardless of the segmentation method. This work on scan/rescan reproducibility builds upon our previous work on studying reproducibility of resting state fMRI, fractional anisotropy, and brain morphology (Shou et al., 2013). Third, we use a blinded randomized reader study to compare segmentation results of SPM and FSL. This provides valuable clinical information about the accuracy of the segmented tissues.

Proper classification of brain tissue plays a crucial role in the statistical analysis of neuroimaging data. Thus, there is an urgent need to understand and quantify the reproducibility of brain segmentation results across software platforms and studies. Our results indicate that: (1) SPM and FSL provide results that exhibit moderate to large differences indicating differences between the two software platforms; (2) there is no statistically significant scan effect; and (3) there is a statistically significant segmentation method effect: significant differences were detected between the two segmentation methods for gray matter and white matter at all probability thresholds considered and for cerebrospinal fluid at two of the three thresholds.

## Materials and methods

### Subjects

The dataset is named Kirby21 (Landman et al., 2011) and is publicly available online at https://www.nitrc.org/projects/multimodal/. Twenty-one healthy volunteers (average age 31.8, *sd* = 9.5, 10 Females), were scanned using multiple imaging techniques including MRI. Local institutional review board approval and written informed consent were obtained prior to examination. Two MRI scans were collected, taken approximately 3 h apart. The sequence parameters for the MPRAGE scans in the Kirby21 dataset (Landman et al., 2011) were as follows; “A 3D inversion recovery sequence was used (TR/TE/TI = 6.7/3.1/842 ms) with a 1.0 × 1.0 × 1.2 mm^3^ resolution over an FOV of 240 × 204 × 256 mm acquired in the sagittal plane. The SENSE acceleration factor was 2 in the right-left direction. Multi-shot fast gradient echo (TFE factor = 240) was used with a 3 s shot interval and the turbo direction being in the slice direction (right-left). The flip angle was 8°. No fat saturation was employed. The total scan time was 5 min 56 s.”

### Image segmentation

Image segmentation for both neuroimaging software tools, as well as for regions of interest, was performed for both MRI scans using identical approaches. Images were processed using two standard neuroimaging packages: Statistical Parametric Mapping version 12 (Penny et al., 2011) implemented in MatLab (MathWorks) and FMRIB Software Library v5.0 (Jenkinson et al., 2012). The FSL package was used via the statistical package R through the FSLR library, a wrapper implemented by John Muschelli (https://cran.r-project.org/web/packages/fslr/index.html). Images were segmented into gray matter (GM), white matter (WM), and cerebrospinal fluid (CSF). A detailed description of segmentation approaches is provided below. Links with our code and generated datasets are provided in the Supplemental Material.

### FSL segmentation

For FSL, images were first bias-field corrected using the N4 algorithm (Tustison et al., 2010) to remove low frequency intensity variations. Images were then skull-stripped using FSL Brain Extraction Tool (BET) (Smith, 2002) with the default parameters. The FAST (FMRIB's Automated Segmentation Tool) algorithm in FSL was used on the N4-normalized skull-stripped images to generate a tissue probability map for three tissue classes: gray matter (GM), white matter (WM), and cerebrospinal fluid (CSF). The result of the FAST algorithm (Zhang et al., 2001) is the relative probability that every voxel is GM, WM, or CSF. Segmentations were performed in the native space (subject space) and not in a standard anatomical space.

To calculate the volume for each tissue class, probability maps were threshold at three levels 0.5, 0.8, and 0.9, each generating a different binary mask. The volume for each tissue class and threshold pair was obtained by multiplying the number of voxels with an assigned probability above a given threshold with the dimension of the voxel. The volume was expressed in mL by dividing this number by 1000. In addition to the volumes computed at different thresholds, a weighted volume, presented in the Supplemental Material, was also calculated by summing the voxel-specific probabilities and multiplying this sum by the dimension of the voxel within each tissue class and dividing by 1000.

### SPM segmentation

Segmentation (via SPM12) combines bias-field correction with segmentation and registration to a standard anatomical space. Unlike FSL, SPM12 does not perform skull stripping before segmentation and uses tissue probability maps as priors for the segmentation (Ashburner and Friston, 2005). SPM segmentation provides classification into six tissue classes (GM, WM, CSF, skull, soft-tissue, and air). In addition to probability maps in the standard anatomical space, SPM provides an inverse deformation field that can be used to generate tissue probability maps in the native space. To calculate the volume for each tissue class, probability maps were threshold at three levels 0.5, 0.8, and 0.9, each generating a different binary mask. Using the same technique described for FSL, the volume for GM, WM, and CSF can be computed.

### Region of interest selection and extraction

**Figure 1:**
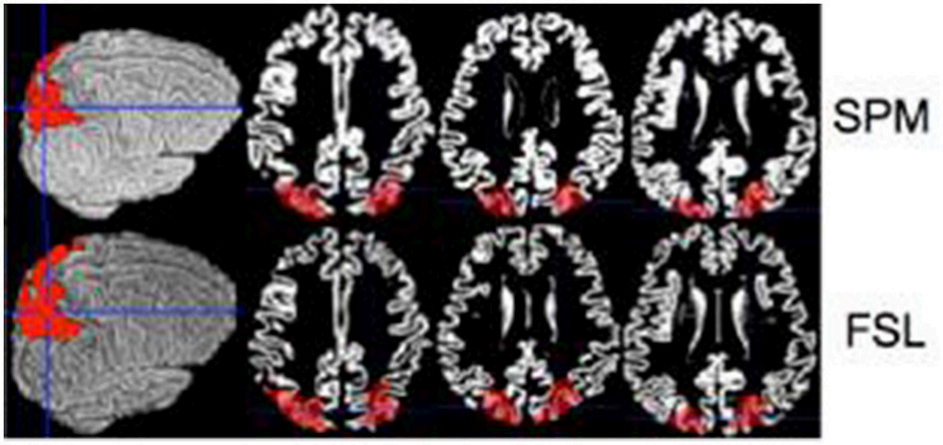
**Inferior parietal ROI is shown on a single brain (native space) for the SPM (top row) and the FSL (bottom row)**.

To quantify differences between SPM and FSL we compared estimated volumes of regions of interest. Ten random gray matter regions of interest (ROI) were selected from the Automated Anatomical Labeling Atlas (Tzourio-Mazoyer et al., 2002), available in SPM through the WFU pickatlas toolbox (Maldjian et al., 2003). The ROIs considered were: anterior cingulate cortex, middle frontal gyrus, superior frontal gyrus, paracentral lobule, parietal inferior, parietal superior, postcentral, precentral, superior motor, temporal superior, all bilateral. Figure 1 displays one of these ROIs (parietal inferior). All ROIs were extracted in the MNI template space. ROIs were then mapped to the native space using the registration approach described in ROI Extraction.

### ROI extraction

#### SPM ROI extraction

Importantly, ROI's were first coregistered to native space and then the volume was calculated. During segmentation in SPM12, for each subject an inverse deformation field is generated that registers every component of the brain (i.e., ROI) in the standard space (MNI) to the native space. This deformation field was applied to all ten ROIs using a nearest-neighbor interpolation. To extract the volume of the ROIs in the native space, probability maps were threshold at the same three probability thresholds used for SPM and the extracted volume was expressed in milliliters.

#### FSL ROI extraction

Similar to SPM, ROI's were first coregistered to native space and then the volumes were calculated. To generate a similar deformation field for FSL, we used FSL to register the ROIs to the native space. The FSL ROI extraction involved the following steps: (1) use an affine registration between the structural MPRAGE and the MNI template, using the function FLIRT (FMRIB's Linear Image Registration Tool) in FSL; (2) use the parameters from FLIRT to conduct a non-linear registration between the structural and the MNI template, using the function FNIRT (FMRIB's Non-linear Registration; Jenkinson and Smith, 2001; Jenkinson et al., 2002) in FSL; and (3) use the FNIRT warp file to coregister the ROI from the template space into each subject's native space, using the applywarp FSL function. Volumes were expressed in mL.

#### Neuroradiology ratings

Each GM, WM, and CSF was rated independently by two neuroradiologists on a scale from 1 to 4 with 1 being poor (incorrect classification of all or a significant portion of an anatomic structure) and 4 being excellent. Results of our ratings study are described in Neuroradiology Ratings. Neuroradiologists were blinded to the image segmentation method. The neuroradiologists have never used SPM or FSL for segmentation and they confirmed that they could not tell which image was from SPM or from FSL. They have used FSL in their previous work on Diffusion Tensor Imaging (DTI).

#### Statistical methods

Descriptive statistics (means, standard deviations) were calculated for all measurements. To assess the differences in the brain volume measurements between the two methods and two scans, a two way repeated measures analysis was performed with two fixed factors (scan and method) and a random subject effect to account for within-subject correlation. The interaction between scan and method was tested but it was not found to be statistically significant in any of the models. Thus, results for the main model include only main effects. The Kenward-Rogers method (Kenward and Roger, 1997) was used for computing the number of degrees of freedom. The following statistical model was fit for each subject's brain tissue type volume (*y*):

$$\begin{array}{l} y_{ij}=\beta_{0}+\beta_{1}M_{ij}+\beta_{2}S_{ij}+b_{i0}+\varepsilon_{ij}, \end{array}$$

where, β_0_ represents the intercept, *M*_*ij*_ is the segmentation method factor (*M* = 0 for FSL, *M* = 1 for SPM) for the *j*^th^ observation on the *i*^th^ subject (*i* = 1, 2, …, 21, *j* = 1, 2 ,…, 4 (= *n*_*i*_) since there are *n*_*i*_ = 4 observations per subject), *S*_*ij*_ is the scan factor (*S* = 0 for scan 1, *S* = 1 for scan 2), *b*_*i*0_ is the subject-specific random effect [$b_{0i}\sim N\left(0,\sigma_{0}^{2}\right)$] , and $\varepsilon_{ij}\sim N\left(0,\sigma_{e}^{2}\right)$ is the random error term. The term β_1_ represents the difference in the tissue volume between methods (SPM vs. FSL) when the scan index is fixed and β_2_ represents the difference in the tissue volume between scan 1 and scan 2 when the method is fixed.

The same statistical model presented above was used for the analysis of each of the calculated tissue type volume, at each threshold as well as for the weighted sum volume and for each ROI (at all thresholds). The repeated measures analysis was performed in SAS 9.3 (SAS Institute, Cary, NC) while the descriptive analysis and plots were performed in R (R Core Team, 2013; http://www.R-project.org/). All statistical tests were two sided and test results were considered significant if the associated *p* < 0.05. No multiple comparison correction was performed.

## Results

### Descriptive statistics: gray matter, white matter, and CSF

The average brain volume for each tissue type across each scan was almost identical within each method, but different between methods (Table 1 in the paper, Figure 1 in the Supplemental Material). The gray matter volume computed using FSL was lower on average than the gray matter volume computed from SPM at all thresholds considered.

**Table 1:** **Descriptive statistics for threshold volumes for each tissue type**.

**Tissue type**	**Scan1 mean (*sd*)**	**Scan2 mean (*sd*)**	**Threshold**	**Scan1 mean (*sd*)**	**Scan2 mean (*sd*)**
***SPM***				***FSL***	
*GM*	983.59 (100.05)	984.14 (96.86)		829.25 (72.81)	826.21 (72.71)
*WM*	595.54 (62.17)	595.81 (58.37)	***Threshold 0***	575.63 (59.10)	575.77 (58.68)
*CSF*	769.16 (146.98)	775.11 (132.12)		462.19 (35.79)	465.09 (37.19)
*GM*	711.57 (66.21)	711.35 (66.42)		566.94 (53.67)	561.90 (54.64)
*WM*	456.28 (49.72)	454.90 (47.28)	***Threshold 0.5***	507.95 (55.74)	507.84 (56.06)
*CSF*	274.60 (73.02)	276.50 (74.33)		270.41 (23.73)	274.76 (27.71)
*GM*	626 (55.82)	624.55 (58.16)		327.50 (34.42)	323.32 (35.05)
*WM*	424.56 (47.42)	423.19 (45.01)	***Threshold 0.8***	384.54 (46.90)	384.19 (47.24)
*CSF*	190.26 (60.58)	190.65 (61.06)		174.55 (17.03)	176.77 (22.12)
*GM*	507.99 (45.13)	504.54 (49.10)		312.36 (32.23)	308.38 (33.12)
*WM*	384.89 (44.51)	383.54 (42.23)	***Threshold 0.95***	358.95 (42.93)	358.88 (43.03)
*CSF*	116.50 (43.70)	115.72 (43.24)		161.87 (17.17)	164.24 (22.63)

The white matter volume computed using FSL was lower than that computed with SPM at all thresholds, except for the 0.5 probability threshold. The CSF volume computed using FSL was lower at probability thresholds of 0.5 and 0.8 than the SPM volume, but higher at the 0.95 probability threshold. Weighted sum probability volumes descriptive statistics and parameter estimates from the repeated measures model are presented in the Supplemental Material (Tables 2, 3).

In addition, we have also computed intra-class correlation coefficients (ICC) using a one-way random effects model between scan 1 and scan 2 for each tissue type volume within each segmentation method and each threshold (Table 7 in Supplemental Material; Shrout and Fleiss, 1979; McGraw and Wong, 1996). The random effects model that was used for the ICC is not for binary data but for continuous data (e.g., GM volume), for each subject and each scan. The ICC was used to quantify the within-method correlation for each tissue type and probability threshold. A high degree of reliability (all ICC's above 0.8) was found between scan 1 and 2 for each tissue type at each threshold within each method.

#### Gray matter

Parameter estimates for each method and individual parameter test results are presented in Table 2. There was no statistically significant effect of scan at any threshold (*p* > 0.05). There was a statistically significant effect of segmentation method for the gray matter volume at all probability thresholds (*p* < 0.0001), with higher volumes on average for the SPM segmentation.

**Table 2:** **Repeated measures analysis results for gray matter by threshold**.

**Threshold**	**Effect**	**β (*SE*)**	***t*(*df*)**	***p*-value**	**95% CI for β**
***GRAY MATTER (GM)***					
*0*	Intercept	828.36 (18.71)	44.28 (21.6)	<0.0001	(789.92, 867.19)
	Method (FSL = ref)	156.13 (5.17)	30.20 (61)	<0.0001	(145.80, 166.47)
	Scan (Scan1 = ref)	−1.24 (5.17)	−0.24 (61)	0.81	(−11.58, 9.09)
*0.5*	Intercept	565.73 (13.10)	43.19 (21.4)	<0.0001	(538.53, 592.93)
	Method (FSL = ref)	147.04 (3.42)	42.89 (61)	<0.0001	(140.19, 153.90)
	Scan (Scan1 = ref)	−2.62 (3.42)	−0.77 (61)	0.44	(−9.47, 4.23)
*0.8*	Intercept	326.82 (10.09)	32.40 (23.6)	<0.0001	(305.98, 347.66)
	Method (FSL = ref)	299.86 (4.07)	73.66 (61)	<0.0001	(291.72, 308)
	Scan (Scan1 = ref)	−2.81 (4.07)	−0.69 (61)	0.49	(−10.96, 5.32)
*0.95*	Intercept	312.23 (8.63)	36.20 (24.7)	<0.0001	(294.45, 330.00)
	Method (FSL = ref)	195.89 (3.89)	50.28 (61)	<0.0001	(188.1, 203.68)
	Scan (Scan1 = ref)	−3.71 (3.89)	−0.95 (61)	0.34	(−11.50, 4.07)

*β coefficient for the method represents the difference in mean estimates between SPM and FSL when scan is fixed; β_0_ from the model equation corresponds to intercept row, β_1_ corresponds to method row, β_2_ corresponds to scan row*.

#### White matter

Parameter estimates for each method and individual parameter test results are presented in Table 3. There was no statistically significant effect of scan at any threshold (*p* > 0.05). There was a statistically significant effect of segmentation method for the white matter volume at all probability thresholds (*p* < 0.0001), with higher volumes on average for the SPM segmentation at 0.8 and 0.95 probability thresholds and lower at the 0.5 threshold.

**Table 3:** **Repeated measures analysis results for white matter by threshold**.

**Threshold**	**Effect**	**β (*SE*)**	***t(df)***	***p*-value**	**95% CI for β**
***WHITE MATTER (WM)***					
*0*	Intercept	575.60 (12.89)	44.65 (21.4)	<0.0001	(548.81, 602.38)
	Method (FSL = ref)	19.97 (3.34)	5.98 (61)	<0.0001	(13.30, 26.65)
	Scan (Scan1 = ref)	0.21 (3.33)	0.06 (61)	0.95	(−6.47, 6.88)
*0.5*	Intercept	508.27 (11.36)	44.71 (20.7)	<0.0001	(484.61, 531.93)
	Method (FSL = ref)	−52.31 (2.14)	−24.44 (61)	<0.0001	(−56.58, −48.03)
	Scan (Scan1 = ref)	−0.74 (2.14)	−0.35 (61)	0.73	(-5.02, 3.54)
*0.8*	Intercept	384.80 (10.13)	37.99 (20.8)	<0.0001	(363.73, 405.88)
	Method (FSL = ref)	39.50 (1.99)	19.77 (61)	<0.0001	(35.51,43.50)
	Scan (Scan1 = ref)	−0.87 (1.99)	−0.43 (61)	0.67	(−4.86, 3.13)
*0.95*	Intercept	359.27 (9.38)	38.30 (20.7)	<0.0001	(339.74, 378.79)
	Method (FSL = ref)	25.30 (1.75)	14.43 (61)	<0.0001	(21.80, 28.81)
	Scan (Scan1 = ref)	−0.71 (1.75)	−0.40 (61)	0.69	(−4.21, 2.80)

*β coefficient for the method represents the difference in mean estimates between SPM and FSL when scan is fixed.; β_0_ from the model equation corresponds to intercept row, β_1_ corresponds to method row, β_2_ corresponds to scan row*.

### Regions of interest

Parameter estimates for all ROIs, each method, and individual parameter test results are presented in the Supplemental Material (4–6).

**Figure 2:**
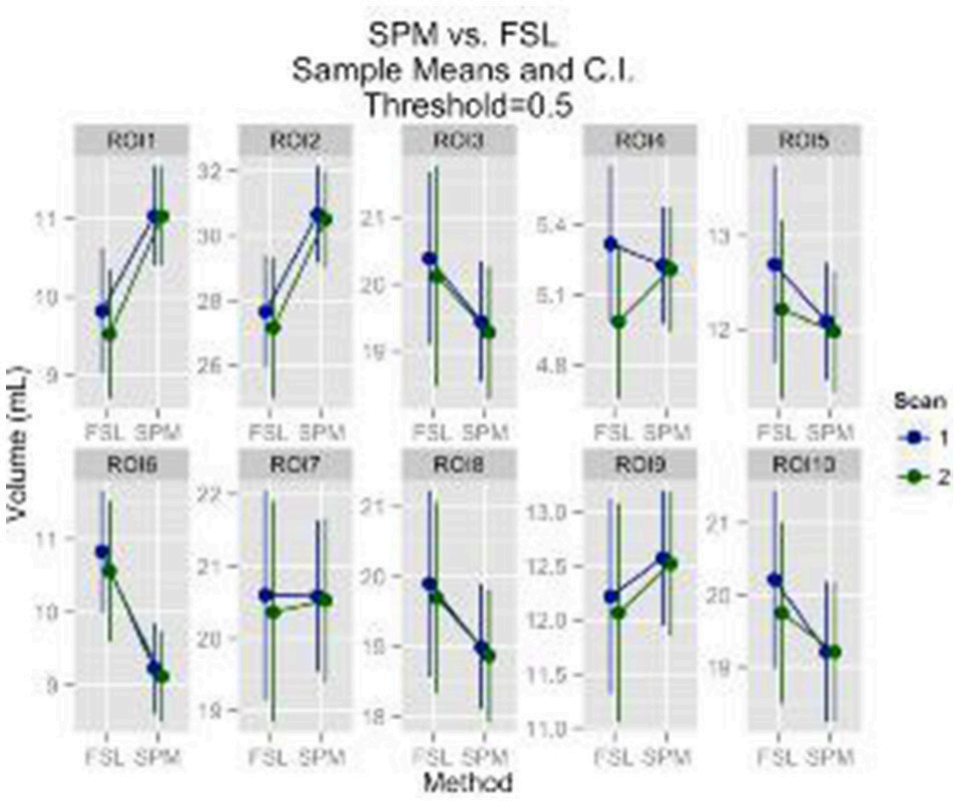
**GM ROI's mean volumes and 95% CI are plotted**. The ROI's are: anterior cingulate cortex, middle frontal gyrus, superior frontal gyrus, paracentral lobule, parietal inferior, parietal superior, postcentral, precentral, superior motor, temporal superior, respectively.

Regions of interest (ROI) analysis results followed the same pattern with results for the gray matter volume. A statistically significant result of scan effect was identified for only 1 out of 10 ROIs at the 0.95 probability threshold (*p* = 0.046), which could very well be due to chance. A statistically significant effect of segmentation method was detected for all ROI's at the probability thresholds 0.8 and 0.95. For the 0.5 probability threshold a statistically significant effect of segmentation method was identified for 6 out of the 10 ROI's. There was no statistically significant effect of scan for any of the ROI's at the 0.8 and 0.5 probability thresholds. The descriptive statistics for the 0.5 probability threshold are provided in Figure 2.

### Neuroradiology ratings

Two neuroradiologists provided good and close performance ratings for gray matter segmentation for the two methods and scans (40 out of 42 were rated excellent for both SPM and FSL by each rater) (Figure 2 in Supplemental Material). In contrast, for white matter segmentation both methods were rated poorly (Figure 3 in Supplemental Material). FSL had a higher percentage of being rated poorly on both scans (42 white matter segmentations out of 42 were rated poorly for FSL compared to 9 for SPM by rater 1; 42 out of 42 rated poorly for FSL compared to 10 out of 42 for SPM by rater 2).

#### Neuroradiology case study

**Figure 3:**
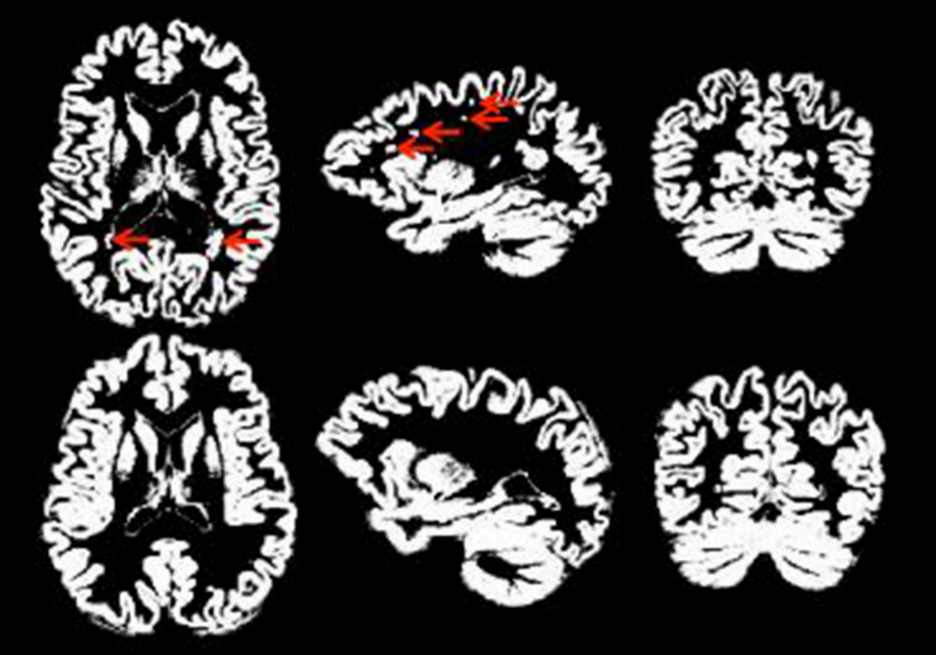
**Good (bottom row) vs. poor (top row) GM segmentation**. Red arrows indicate regions that are problematic/incorrectly classified as gray matter.

**Figure 4:**
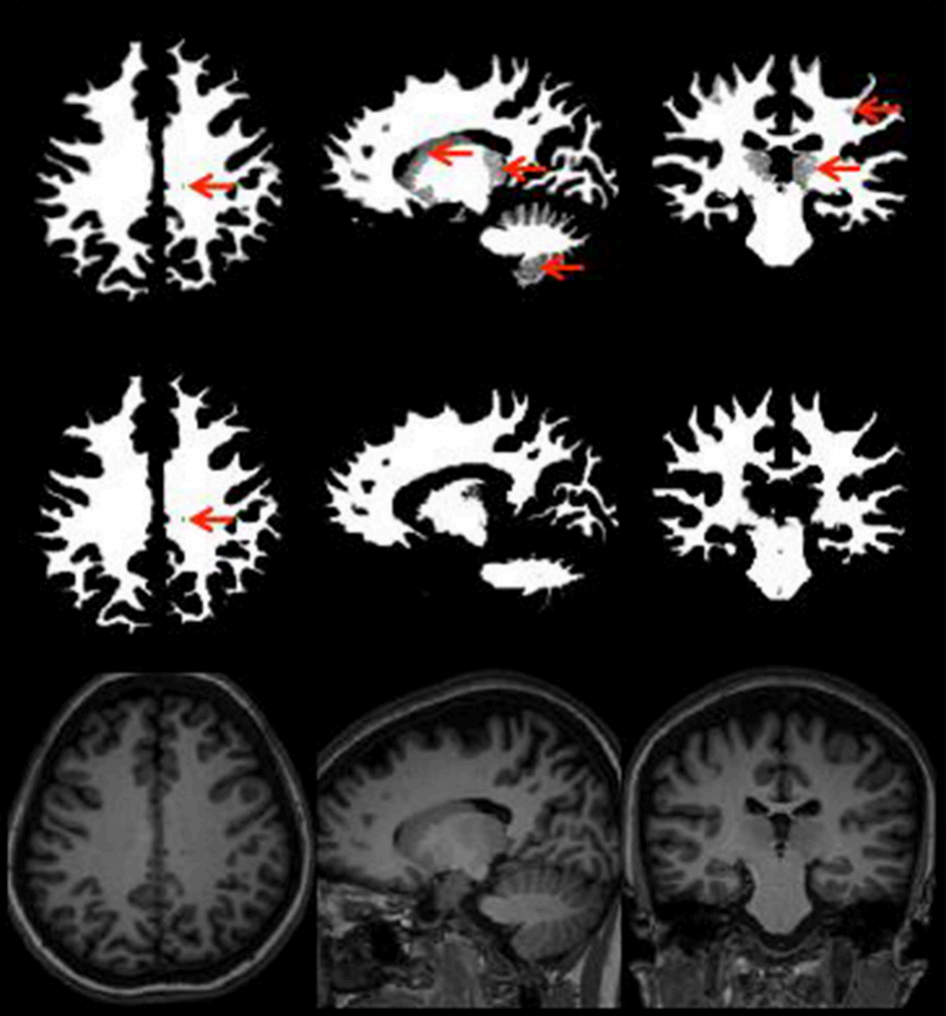
**White matter segmentation with the areas poorly rated (top row), rated as good (middle row), and MPRAGE of the same subject (bottom row) showing the same slice white matter without any potential problems**. Red arrows point to areas of potential issues.

Figure 3 provides an illustration of neuroradiology ratings of gray matter tissue segmentation that was rated excellent vs. one that was rated poorly. Red arrows indicate areas where the tissue was not properly classified. Figure 4 provides a similar contrast for white matter. The original MPRAGE is displayed on the last row of Figure 4.

Additional examples with segmentation issues are presented and discussed in the Supplemental Material (Figures 5–10 in Supplemental Material). These case studies seem to indicate that BET could sometimes affect the follow-up segmentation algorithm and that FSL may have more problems differentiating white from gray matter in sub-cortical regions even when BET performs skull stripping well.

## Discussion

Artifacts and partial volume effects can affect brain tissue segmentation. In this paper, we compared SPM and FSL segmentation methods and we focused on: (1) differences between the segmentation methods; (2) reliability of the segmentations across two scans taken a few hours apart; and (3) randomized reader studies to compare the perceived quality of segmentation by clinical neuroradiologists. We found that there were moderate to large differences between segmentation platforms and a strong within-subject, within-platform reliability. We have also found that clinical neuroradiologists agree that: SPM and FSL perform well in gray matter segmentation; and SPM and FSL both perform poorly in white matter segmentation.

MRBrainS is an imaging computational challenge started at Landman et al. (2012) dedicated to comparing the performance of segmentation of gray matter, white matter and cerebrospinal fluid on multi-sequence (T1-weighted, T1-weighted inversion recovery and FLAIR) 3 Tesla MRI scans of the brain (http://mrbrains13.isi.uu.nl/). MRBrainS compared various methods among themselves, and relative to a ranking system (Mendrik et al., 2015). Using manual segmentations as the ground truth, results indicate that SPM and FSL performed worse than other algorithms, and that SPM seems to outperform FSL in overall ranking as well as gray/white matter segmentation (Mendrik et al., 2015). Several other previous studies have also compared SPM and FSL segmentations. Tsang et al. found that SPM5's segmentation performed more accurately than FSL segmentation (Tsang et al., 2008). Kazemi and Noorizadeh (2014) reported that SPM8's segmentation performed better compared to FSL in the presence of noise. Klauschen et al. (2009) reported significant differences in gray matter volume and white matter volume between SPM and FSL. Our results complement these studies; they indicate that there are differences between the methods; that these differences are not due to software reliability, that results can differ dramatically with the probability threshold used, and that SPM and FSL perform quite differently for different tissue classes in terms of perceived clinical accuracy. Our results add that SPM and FSL perform similarly within subject across two scans indicating that these methods are robust to between scan factors. Similar to previous studies (Mendrik et al., 2015), we found that the perceived differences between FSL and SPM (see Supplemental Material from neuroradiologists in Neuroradiology Case Study) were due to FSL's inability to accurately distinguish between deep cortical gray matter and white matter.

Results indicate that there are significant differences in gray matter, white matter, and CSF segmentations between SPM and FSL. They indicate that differences may be due to the segmentation approach, but the choice of probability thresholds may have a much larger impact on results. While sensitivity in the probability thresholds is expected, the large effect of these thresholds has been under-reported.

A study of threshold comparison between SPM and FSL may reduce observed differences between results. Investigating if such equivalent probability thresholds exist, one would need to study if the relationships are preserved across subjects, tissue classes, and regions of interest.

Results indicate that the reported effects are both global and local. Indeed, ROI volumes results mirrored whole brain results for different regions of the brain. In general, differences between scans are negligible when compared to differences between platforms and probability thresholds.

We also identified strong within-subject reliability of segmentation, though reliability is only a part of the story. Indeed, it is actually worse to produce reliably poor results than to produce unreliably good results. As there are large differences in results between platforms and probability thresholds, we conclude that either or both methods are biased.

Our study indicates that the differences found between SPM and FSL tissue volumes computed from the segmentations depend in a complex way on the various tuning parameters associated with individual segmentation steps of each algorithm. FSL relies only on image intensity to conduct segmentation, which may be more prone to inaccurately segment parts of the gray matter as white matter. This is likely due to heterogeneity across images as well as overlap between gray and white matter intensities in certain images. We have noticed that this occurs in several subcortical structures (e.g., caudate/thalamus), which seems to support the hypothesis that such substructures are more likely to exhibit white-gray matter intensity overlap. Moreover, the scatter plots of white and gray matter intensities do not indicate perfect separation in intensities. Thus, irrespective to the performance of the clustering algorithm used by FSL, it remains more difficult to segment gray matter from white matter. In contrast, SPM uses both image intensity and spatial prior information, which may be the reason for improved segmentation. For example, in situations where registration to a template is decent, SPM's spatial priors provide information about where the caudate is, which helps segmentation. This suggests that: (1) at least for healthy brains that register relatively well to the SPM template, the spatial priors contain additional information; (2) in un-healthy brains that have sizeable pathology and deformation SPM may actually induce bias and perform worse than FSL or other methods; and (3) improved spatial registration, such as multi-atlas label fusion, and population-specific templates may improve performance of segmentation algorithms. One approach to test this is to perform manual segmentations of individual ROIs, such as the caudate, and compare them to white/gray matter segmentation algorithms in these sub-structures. Another possible explanation could be that skull-stripping using BET in FSL may have an effect on segmentation. One approach to testing whether BET reliably segments the skull is to perform BET on multiple subjects with hand segmentation of the brain. As we perform manual segmentation on many images acquired in our lab, such a study could be performed on a relatively large population of older individuals. This could give insight as to how accurate/inaccurate BET is, but it could also reveal where in the brain BET is inaccurate.

Our study has several limitations. The sample size of the study was small and the archival study has multiple scans taken a few hours apart, but no ground truth segmentations were available. The randomized reader study provides additional insight into when SPM or FSL perform better and are more useful.

Kirby21 is an archival dataset that collected high-resolution structural MRI images over a short period of time (within the same day). Previous studies (Tsang et al., 2008; Klauschen et al., 2009; Kazemi and Noorizadeh, 2014) have looked at the differences between these methods, however few (Morey et al., 2010) have investigated the reliability of a single segmentation within the same subject for scans taken only hours apart. Importantly, there seems to be a high within platform reliability: segmentations of scans that were only hours apart yielded very similar segmentations and volumes. This may indicate that probability thresholds may have a much bigger effect than previously reported. This may suggest that the interpretation of probabilities or their calculation may be different across platforms. This suggests that estimating a universal correspondence function between the SPM and FSL probability thresholds may reduce the discrepancy between results.

## Author contributions

Analyzed the data: DT, HK, JMM. Contributed analysis tools: JM. Wrote the paper: DT, HK, CC. Discussed the analysis and results: DT, HK, HA, CC. Segmentation ratings: LA, SF.

### Conflict of interest statement

The authors declare that the research was conducted in the absence of any commercial or financial relationships that could be construed as a potential conflict of interest.
