# Supplementary material for: Reproducibility and Bias in Healthy Brain Segmentation: Comparison of Two Popular Neuroimaging Platforms
Source: Front Neurosci. 2016 Nov 9;10:503. doi: 10.3389/fnins.2016.00503 (PMC5101202; doi:10.3389/fnins.2016.00503)
Supplement: Supplementary file 1 [file Presentation1.PDF]

Supplementary Material for *Reproducibility and bias in healthy brain segmentation: comparison of two popular neuroimaging platforms*

Dana L. Tudorascu[1,2,3], Helmet Karim[4], Jacob Maronge[5], Lea Alhilali[6], Saeed Fakhran[7], Howard J Aizenstein[3,4], John Muschelli[8], Ciprian Crainiceanu[8]

[1] Department of Internal Medicine, University of Pittsburgh, Pittsburgh, PA, 15213
[2]Department of Biostatistics, University of Pittsburgh, Pittsburgh, PA, 15213
[3]Department of Psychiatry, University of Pittsburgh, Pittsburgh, PA, 15213
[4]Department of Biomedical Engineering, University of Pittsburgh, Pittsburgh, PA, 15213
[5]Biostatistics Program, Louisiana State University Health Sciences Center, New Orleans, LA, 70112
[6]Department of Neuroradiology, Barrow Neurological Institute, Phoenix, AZ, 85013
[7]Department of Radiology, Banner Health and Hospital Systems, Mesa, AZ, 85202
[8]Department of Biostatistics, Bloomberg School of Public Health, John Hopkins University, Baltimore, MD

Figure 1: Descriptive statistics for each volume by method and threshold

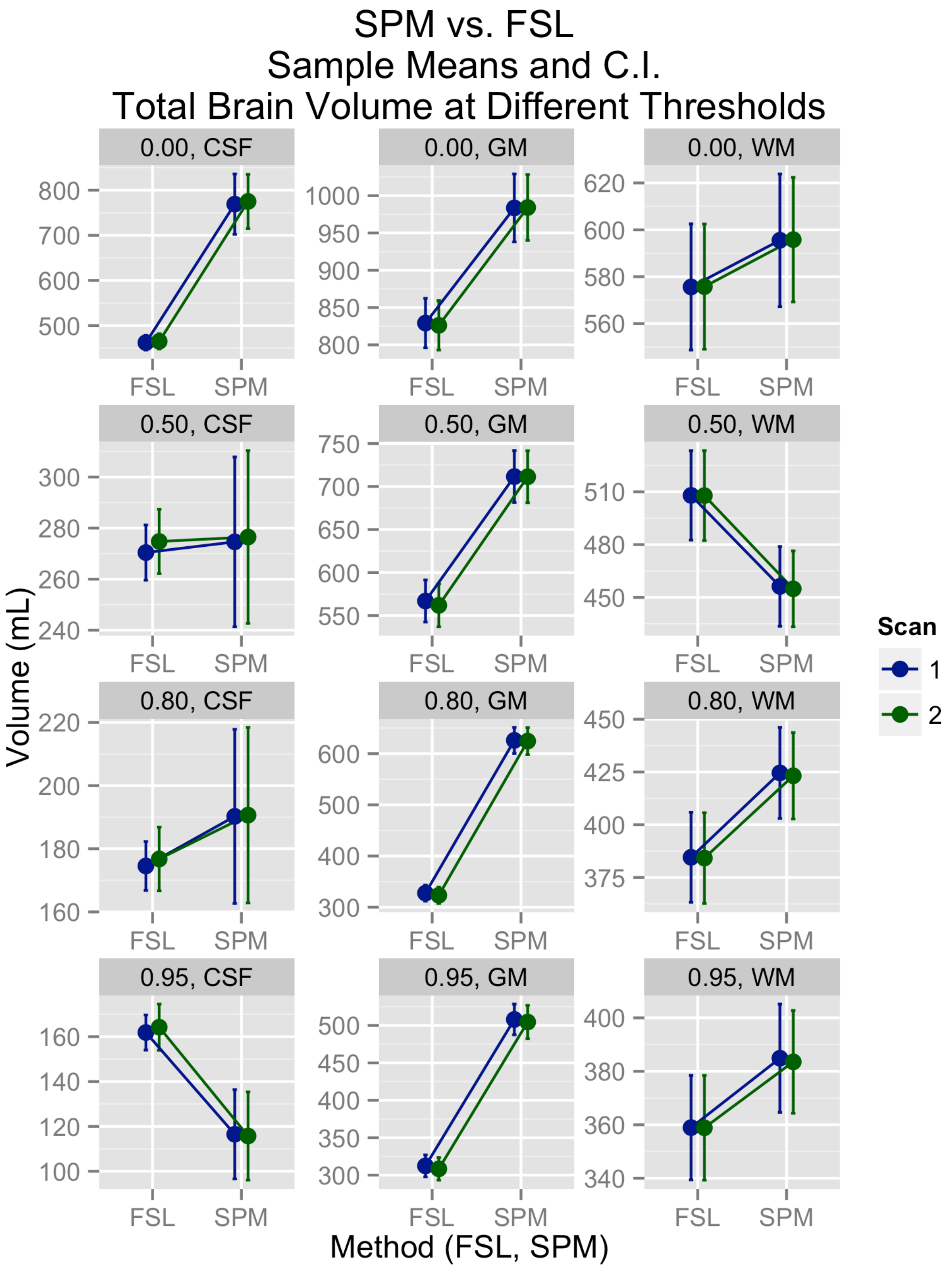

Table 1: Repeated measures analysis results for cerebrospinal tissue by threshold

Threshold	Effect	β (SE)	T (df)	p-value	95% CI for β
**Cerebrospinal Fluid (CSF)**					
0	Intercept	461.43 (20.94)	22.03 (34.5)	<0.0001	(418.90, 503.97)
	Method (FSL=ref)	308.49 (14.89)	20.72 (61)	<0.0001	(278.72, 338.27)
	Scan (Scan1=ref)	4.43 (14.89)	0.30 (61)	0.77	(-25.35, 34.20)
0.5	Intercept	271.02 (11.25)	24.07 (36.4)	<0.0001	(248.20, 293.85)
	Method (FSL=ref)	2.97 (8.36)	0.35 (61)	0.73	(-13.75, 19.68)
	Scan (Scan1=ref)	3.12 (8.35)	0.37 (61)	0.71	(-13.59, 19.83)
0.8	Intercept	175.01 (9.25)	18.92 (35.3)	<0.0001	(156.23, 193.78)
	Method (FSL=ref)	14.79 (6.70)	2.21 (61)	0.03	(1.40, 28.19)
	Scan (Scan1=ref)	1.30 (6.70)	0.19 (61)	0.84	(-12.09, 14.70)
0.95	Intercept	162.65 (7.00)	23.22 (31.8)	<0.0001	(148.38, 176.92)
	Method (FSL=ref)	-46.94 (4.60)	-10.19 (61)	<0.0001	(-56.15, -37.73)
	Scan (Scan1=ref)	0.80 (4.60)	0.17 (61)	0.86	(-8.40, 10.13)

β coefficient for the method *represents the difference in mean estimates between SPM and FSL when scan is fixed;* β_0 from the model equation corresponds to intercept row, β_1 corresponds to method row, β_2 corresponds to scan row.

Figure 2: Neuroradiology ratings for GM

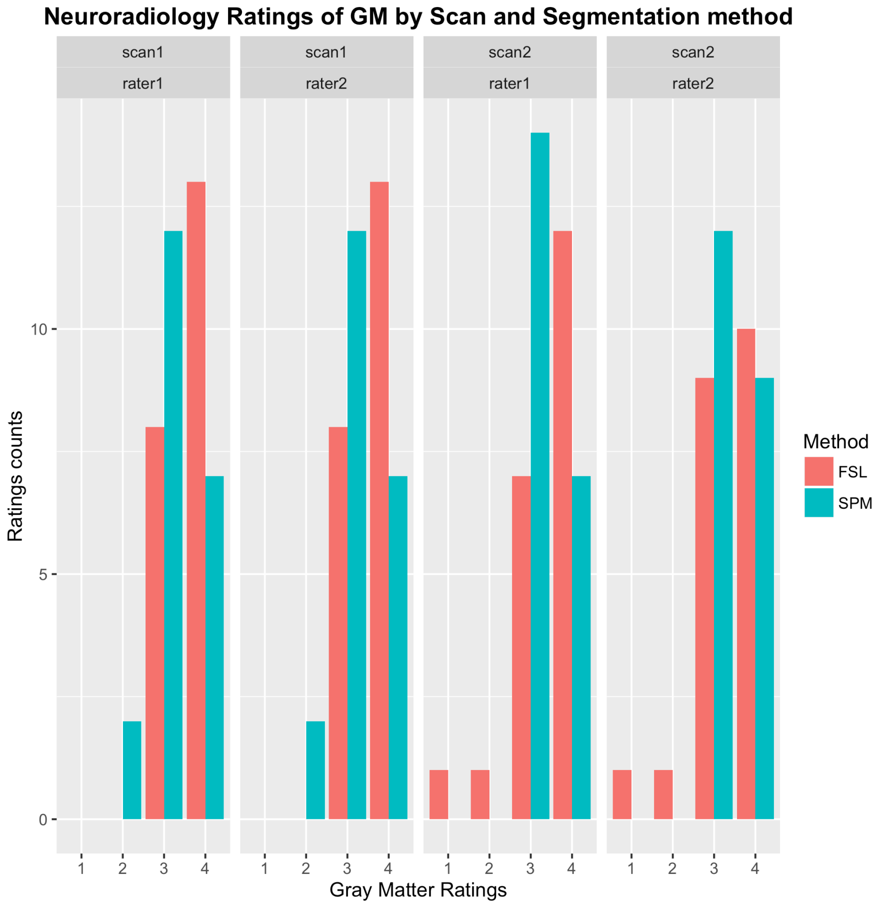

Legend: Side by side plot of neuroradiology ratings for gray matter segmentation for each scan/rater. The x-axes represent the rating and the y-axes represent the frequency of the ratings (i.e. number of subjects in that specific rating category).

Figure 3: Neuroradiology ratings for WM

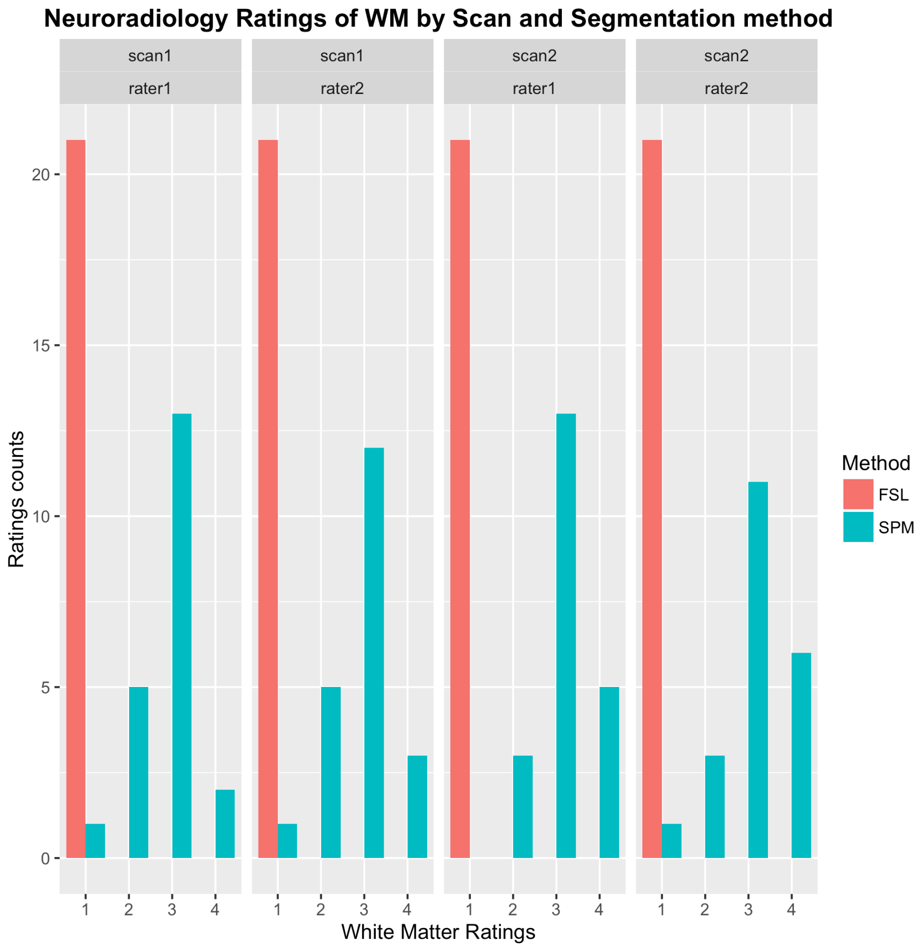

Legend: Side by side plot of neuroradiology ratings for white matter segmentation for each scan/rater. The x-axes represent the rating and the y-axes represent the frequency of the ratings (i.e. number of subjects in that specific rating category).

Figure 4: Neuroradiology ratings for CSF

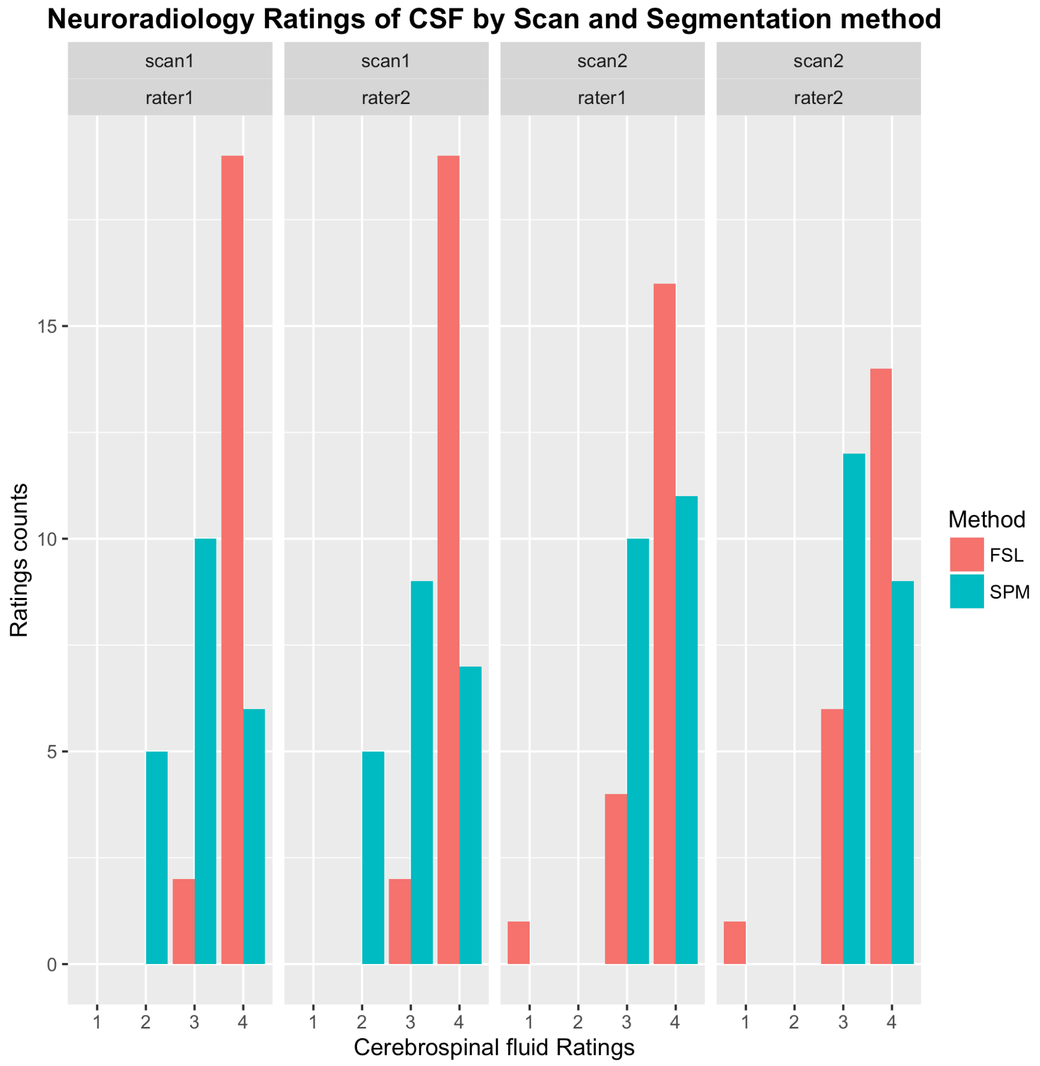

Legend: Side by side plot of neuroradiology ratings for cerebrospinal fluid segmentation for each scan/rater. The x-axes represent the rating and the y-axes represent the frequency of the ratings (i.e. number of subjects in that specific rating category).

Neuroradiology Case studies additional results: Overall, the greatest limitation of the FSL segmentation was a failure to distinguish the deep gray structures from white matter. Often the caudate head and thalamus were included in the white matter segmentation (Figure 5 left, red arrows). SPM was able to successfully exclude these deep gray structures from the white matter segmentation (Figure 5 right, blue arrowheads). Additionally, FSL tended to have difficulty where sulci were small and gyrii were in close proximity, such as the cerebellar folia, where the cortex was often included in the white matter segmentation (Figure 6 (left), red arrows). Similarly, in regions of tightly apposed cortical gyrii, FSL also tended to include the cortex among the white matter segmentation (Figure 7 (left), red arrows). SPM did not include these regions of cortex in the white matter segmentation (Figure 6 (right) and Figure 7 (right), blue arrowheads).

Figure 5: FSL (left) and SPM (right) white matter segmentation (same slice, same subject).

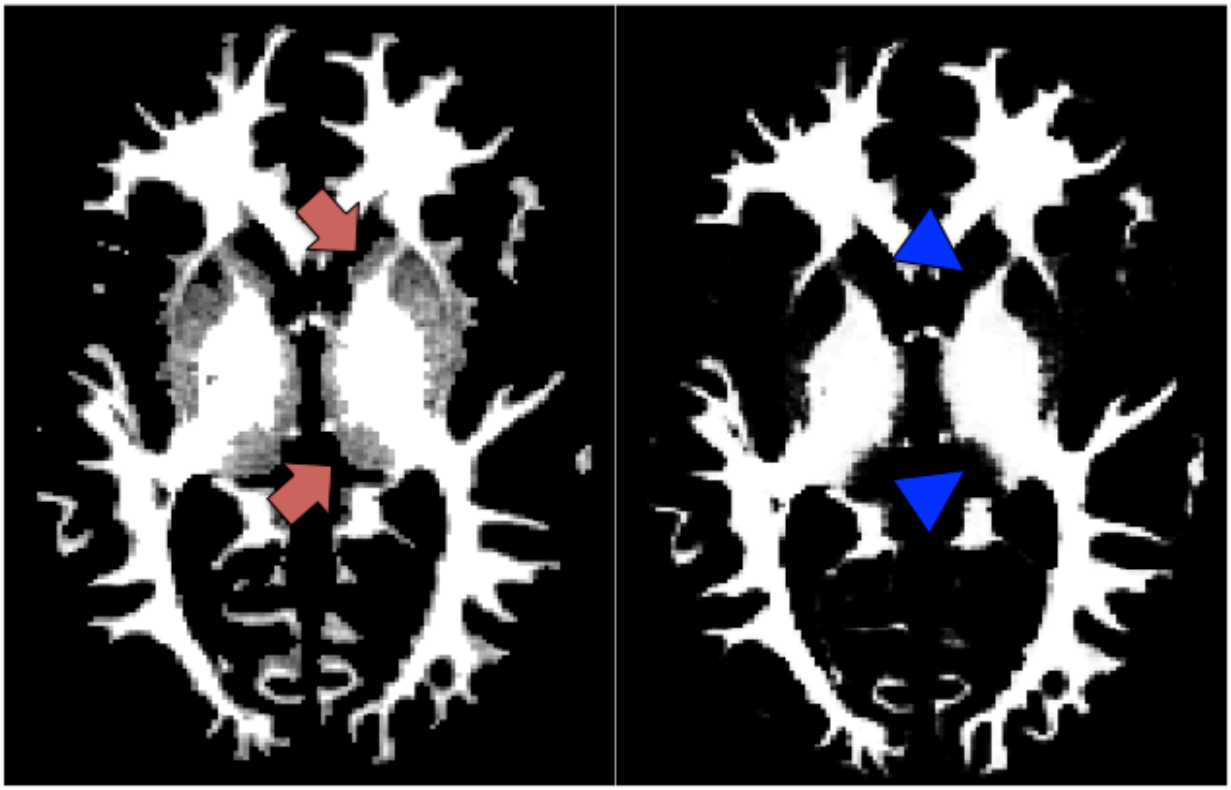

Figure 6: FSL (left) and SPM (right) white matter segmentation (same slice, same subject).

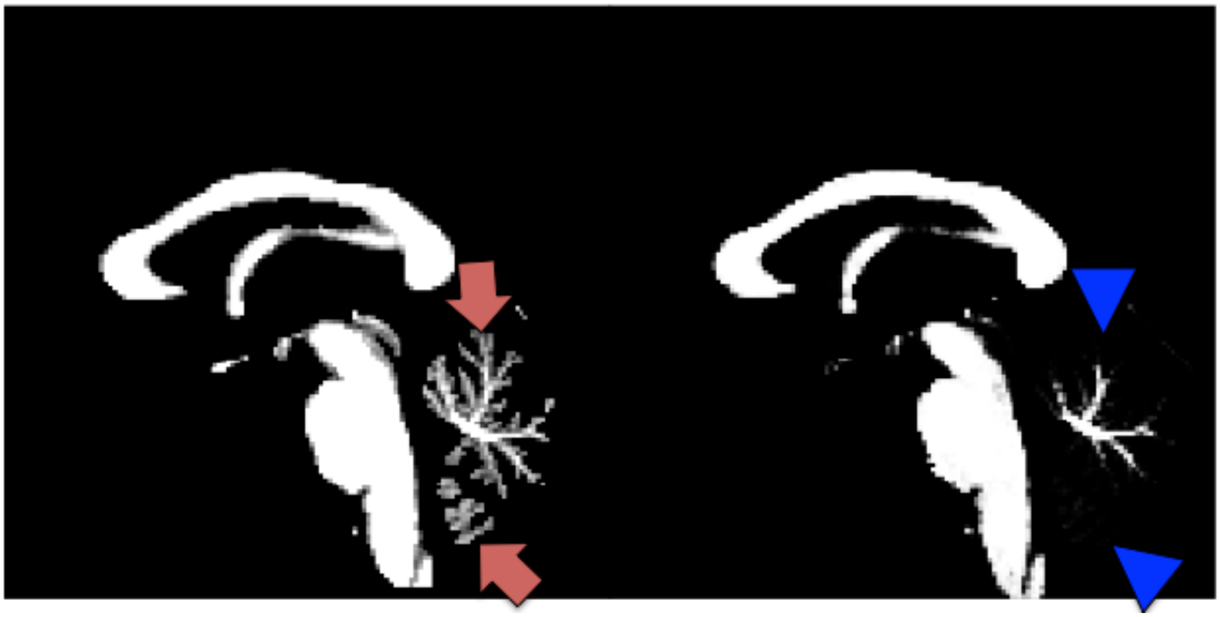

Figure 7: FSL (left) and SPM (right) white matter segmentation (same slice, same subject).

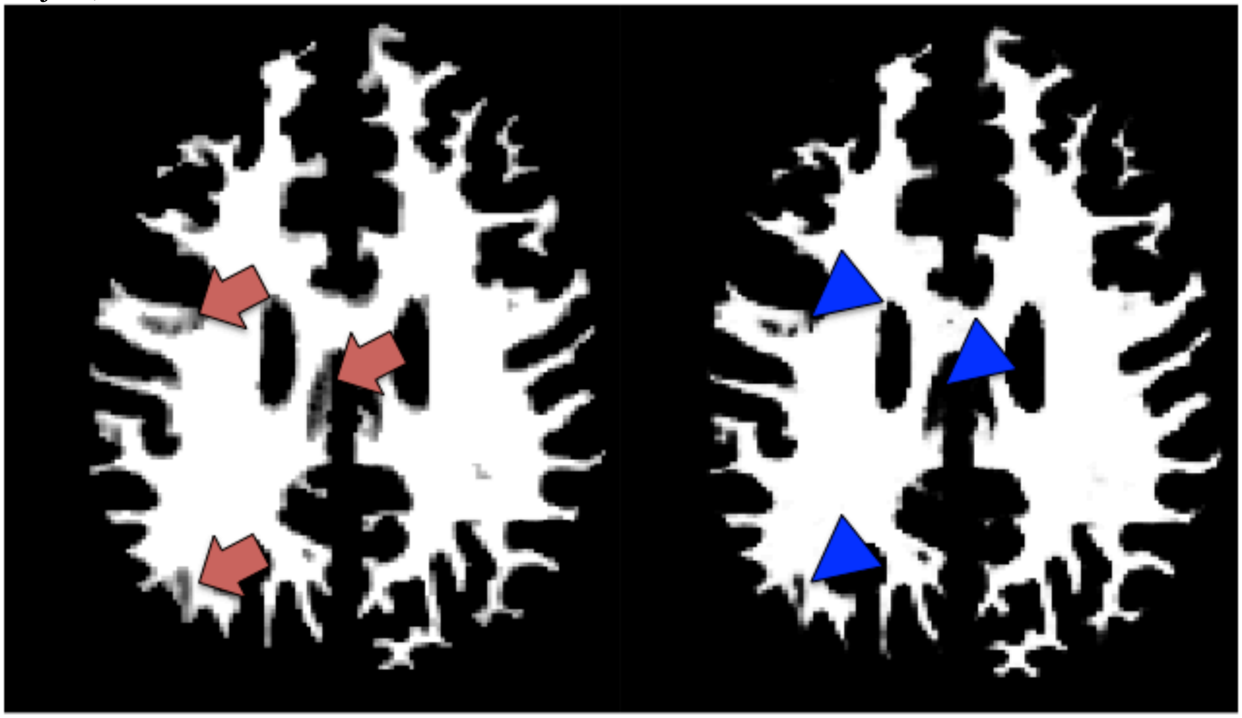

Figure 8: FSL (left) versus SPM (right) same subject (and slice) with red arrows pointing to the areas where GM differences are in the deeper cortical structures;

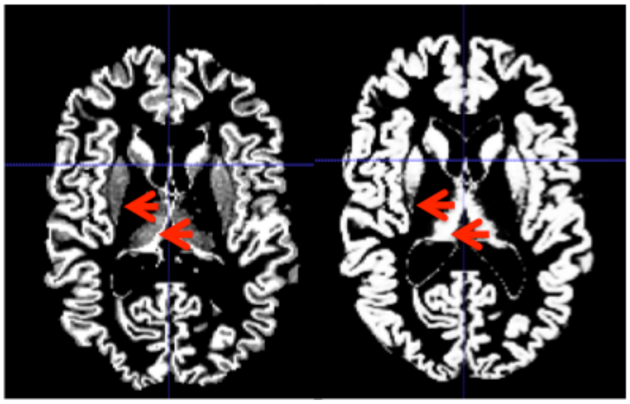

Figure 9: FSL (left) versus SPM (right) same subject (and slice) with red arrows pointing to the areas where GM differences are in the higher cortical structures;

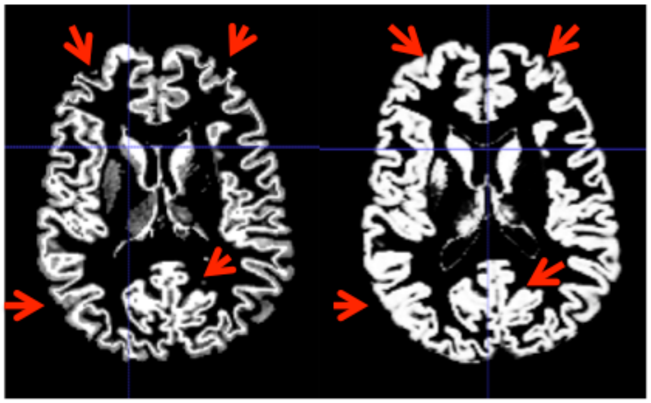

Figure 10: FSL (left) versus SPM (right) same subject (and slice) with red/blue arrows pointing to the areas where CSF differences are;

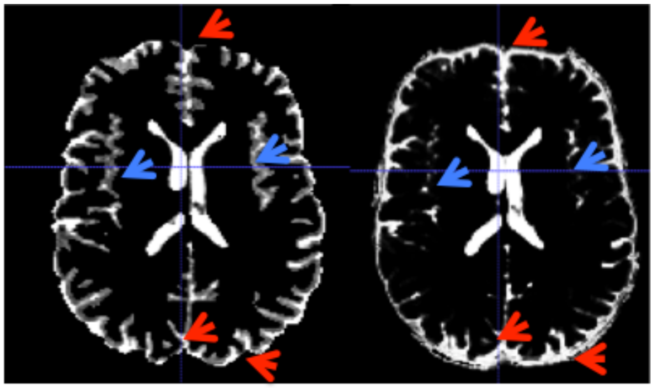

Figure 10 shows that FSL includes more CSF (in the upper cortical (blue arrows)) and SPM does not. This could contribute to the increased GM volume as we have seen in SPM compared to FSL.

Table 2: Weighted volumes summary statistics

Tissue type	Scan1 Mean (sd)	Scan2 Mean (sd)	Scan1 Mean (sd)	Scan2 Mean (sd)
SPM			***FSL***	
GM	703.10 (63.93)	702.58 (64.56)	559.19 (51.01)	555.19 (51.42)
WM	455.01 (49.61)	453.74 (47.00)	486.17 (53.46)	486.06 (53.49)
CSF	289.48 (70.21)	291.74 (71.60)	299.95 (23.66)	303.26 (26.87)

Table 3: Repeated measures analysis results for weighted volumes

Threshold	Effect	β (SE)	T (df)	p-value	95% CI for β
GM	Intercept	558.32 (12.57)	44.40 (21.3)	<0.0001	(532.19, 584.44)
	Method (FSL=ref)	145.66 (3.17)	45.90 (61)	<0.0001	(139.31, 152.00)
	Scan (Scan1=ref)	-2.26 (3.17)	-0.71 (61)	0.48	(-8.61, 4.09)
WM	Intercept	486.45 (11.07)	43.94 (20.7)	<0.0001	(463.42, 509.49)
	Method (FSL=ref)	-31.73 (2.10)	-15.11 (61)	<0.0001	(-35.93, -27.54)
	Scan (Scan1=ref)	-0.69 (2.10)	-0.33 (61)	0.74	(-4.89, 3.51)
CSF	Intercept	300.21 (10.90)	27.55 (35.1)	<0.0001	(278.09, 322.33)
	Method (FSL=ref)	-10.99 (7.86)	-1.40 (61)	0.17	(-26.72, 4.73)
	Scan (Scan1=ref)	2.79 (7.86)	0.35 (61)	0.72	(-12.94, 18.51)

β coefficient for the method *represents the difference in mean estimates between SPM and FSL when scan is fixed;* β_0 from the model equation corresponds to intercept row, β_1 corresponds to method row, β_2 corresponds to scan row.

Table 4: Repeated measures analysis results for each ROI at threshold =0.50

Threshold	Effect	β (SE)	t(df)	p-value	95% CI for β
			Threshold 0.5		
ROI 1	Intercept	9.60 (0.33)	29.02(25.3)	<0.0001	(9.06,10.43)
	Method (FSL=ref)	1.36 (0.16)	8.65 (61)	*<0.0001*	(1.04, 1.67)
	Scan (Scan1=ref)	-0.15 (0.16)	-0.94 (61)	0.35	(-0.46, 0.17)
ROI 2	Intercept	27.57 (0.78)	35.13 (29)	<0.0001	(25.97, 29.18)
	Method (FSL=ref)	3.17 (0.47)	6.81 (61)	*<0.0001*	(2.24,4.10)
	Scan (Scan1=ref)	-0.32 (0.47)	-0.69 (61)	0.49	(-1.25, 0.60)
**ROI 3**	Intercept	20.36 (0.56)	36.55 (29.8)	<0.0001	(19.22, 21.50)
	Method (FSL=ref)	-0.89 (0.34)	-2.62(61)	*0.01*	(-1.58, -0.21)
	Scan (Scan1=ref)	-0.22 (0.34)	-0.64 (61)	0.53	(-0.90, 0.46)
ROI 4	Intercept	5.24 (0.13)	40.35 (40.7)	<0.0001	(4.98, 5.50)
	Method (FSL=ref)	0.07 (0.10)	0.63 (61)	0.53	(-0.14, 0.27)
	Scan (Scan1=ref)	-0.18 (0.10)	-1.68 (61)	0.10	(-0.39, 0.03)
**ROI 5**	Intercept	12.60 (0.38)	33.26 (28.4)	<0.0001	(11.82, 13.37)
	Method (FSL=ref)	-0.42 (0.22)	-1.94 (61)	0.06	(-0.86, 0.01)
	Scan (Scan1=ref)	-0.29 (0.22)	-1.35 (61)	0.18	(-0.73, 0.14)
ROI 6	Intercept	10.79 (0.34)	31.94 (38.8)	<0.0001	(10.10, 11.47)
	Method (FSL=ref)	-1.52 (0.26)	-5.78 (61)	*<0.0001*	(-2.04, -0.99)
	Scan (Scan1=ref)	-0.19 (0.26)	-0.72 (61)	0.47	(-0.72, 0.34)
**ROI 7**	Intercept	20.56 (0.60)	34.37 (25.9)	<0.0001	(19.33, 21.79)
	Method (FSL=ref)	0.07 (0.30)	0.25 (61)	0.81	(-0.52, 0.67)
	Scan (Scan1=ref)	-0.15 (0.30)	-0.49 (61)	0.62	(-0.74, 0.45)

ROI 8	Intercept	19.88 (0.53)	37.73 (26.1)	<0.0001	(18.80, 20.97)
	Method (FSL=ref)	-0.87 (0.27)	-3.27 (61)	*0.002*	(-1.40, -0.34)
	Scan (Scan1=ref)	-0.17 (0.27)	-0.62 (61)	0.53	(-0.70, 0.37)
**ROI 9**	Intercept	12.19 (0.37)	32.99 (27.7)	<0.0001	(11.43, 12.95)
	Method (FSL=ref)	0.41 (0.21)	1.98 (61)	0.052	(-0.005, 0.82)
	Scan (Scan1=ref)	-0.10 (0.21)	-0.50 (61)	0.62	(-0.51, 0.31)
ROI 10	Intercept	20.10 (0.51)	39.21 (25.5)	<0.0001	(19.05, 21.16)
	Method (FSL=ref)	-0.77 (0.25)	-3.13 (61)	0.003	(-1.27, -0.28)
	Scan (Scan1=ref)	-0.23 (0.25)	-0.94 (61)	0.35	(-0.73, 0.26)

β coefficient for the method *represents the difference in mean estimates between SPM and FSL when scan is fixed;* β_0 from the model equation corresponds to intercept row, β_1 corresponds to method row, β_2 corresponds to scan row.

Table 5: Repeated measures analysis results for each ROI at threshold =0.80

Threshold	Effect	β (SE)	t(df)	p-value	95% CI for β
			Threshold 0.80		
ROI 1	Intercept	5.13 (0.27)	19.25 (29.2)	<0.0001	(4.58, 5.67)
	Method (FSL=ref)	4.92 (0.16)	30.93 (61)	*<0.0001*	(4.60, 5.24)
	Scan (Scan1=ref)	-0.10 (0.16)	-0.66 (61)	0.51	(-0.42, 0.21)
ROI 2	Intercept	13.38 (0.50)	26.69 (28.4)	<0.0001	(12.35, 14.40)
	Method (FSL=ref)	12.29 (0.29)	42.53 (41)	*<0.0001*	(11.71, 12.86)
	Scan (Scan1=ref)	-0.19 (0.29)	-0.66 (61)	0.51	(-0.76,0.39)
**ROI 3**	Intercept	9.82 (0.33)	29.52 (29.6)	<0.0001	(9.14, 10.50)
	Method (FSL=ref)	6.15 (0.20)	30.43 (61)	*<0.0001*	(5.75, 6.56)
	Scan (Scan1=ref)	-0.15 (0.20)	-0.73 (61)	0.47	(-0.55, 0.26)
ROI 4	Intercept	3.18 (0.09)	34.17 (37.9)	<0.0001	(2.99, 3.37)
	Method (FSL=ref)	1.23 (0.07)	17.24 (61)	<0.0001	(1.09, 1.37)
	Scan (Scan1=ref)	-0.14 (0.07)	-1.96 (61)	0.055	(-0.28, 0.003)
**ROI 5**	Intercept	6.57 (0.25)	26.30 (26.2)	<0.0001	(6.06, 7.09)
	Method (FSL=ref)	3.49 (0.13)	27.44 (61)	<0.0001	(3.23, 3.74)
	Scan (Scan1=ref)	-0.23 (0.13)	-1.85 (61)	0.07	(-0.49, 0.02)
ROI 6	Intercept	5.78 (0.23)	25.65 (37)	<0.0001	(5.33, 6.24)
	Method (FSL=ref)	1.48 (0.17)	8.74 (61)	*<0.0001*	(1.14, 1.82)
	Scan (Scan1=ref)	-0.14 (0.17)	-0.84 (61)	0.40	(-0.48, 0.20)
**ROI 7**	Intercept	11.78 (0.43)	27.54 (24.1)	<0.0001	(10.90, 12.66)
	Method (FSL=ref)	5.42 (0.18)	29.78 (61)	<0.0001	(5.05, 5.78)
	Scan (Scan1=ref)	-0.18 (0.18)	-1.01 (61)	0.32	(-0.55, 0.18)

ROI 8	Intercept	11.24 (0.38)	29.90 (25.9)	<0.0001	(10.47, 12.01)
	Method (FSL=ref)	4.95 (0.19)	26.35 (61)	*<0.0001*	(4.57, 5.33)
	Scan (Scan1=ref)	-0.21 (0.19)	-1.11 (61)	0.27	(-0.58, 0.17)
**ROI 9**	Intercept	6.56 (0.25)	26.56 (27.5)	<0.0001	(6.06, 7.07)
	Method (FSL=ref)	4.18 (0.14)	30.81 (61)	*<0.0001*	(3.91, 4.45)
	Scan (Scan1=ref)	-0.10 (0.14)	-0.75 (61)	0.45	(-0.37, 0.17)
ROI 10	Intercept	10.97 (0.36)	30.16 (26.8)	<0.0001	(10.22, 11.71)
	Method (FSL=ref)	6.37 (0.19)	33.13 (61)	<0.0001	(5.98, 6.76)
	Scan (Scan1=ref)	-0.20 (0.19)	-1.06 (61)	0.29	(-0.59, 0.18)

β coefficient for the method *represents the difference in mean estimates between SPM and FSL when scan is fixed;* β_0 from the model equation corresponds to intercept row, β_1 corresponds to method row, β_2 corresponds to scan row.

Table 6: Repeated measures analysis results for each ROI at threshold =0.95

Threshold	Effect	β (SE)	t(df)	p-value	95% CI for β
		Threshold 0.95			
ROI 1	Intercept	4.89 (0.25)	19.27 (26)	<0.0001	(4.37, 5.41)
	Method (FSL=ref)	3.48 (0.13)	27.45 (61)	*<0.0001*	*(3.23, 3.74)*
	Scan (Scan1=ref)	-0.14 (0.13)	-1.13 (61)	0.26	(-0.40, 0.11)
ROI 2	Intercept	12.98 (0.47)	27.68 (32.9)	<0.0001	(12.03, 13.94)
	Method (FSL=ref)	5.87 (0.32)	18.40 (61)	*<0.0001*	(5.23, 6.51)
	Scan (Scan1=ref)	-0.23 (0.32)	-0.72 (61)	0.47	(-0.87, 0.41)
**ROI 3**	Intercept	9.50 (0.31)	30.64 (36.7)	<0.0001	(8.87, 10.13)
	Method (FSL=ref)	1.98 (0.23)	8.56 (61)	*<0.0001*	(1.52, 2.45)
	Scan (Scan1=ref)	-0.16 (0.23)	-0.67 (61)	0.50	(-0.62, 0.31)
ROI 4	Intercept	3.00 (0.08)	35.76 (41.7)	<0.0001	(2.84, 3.17)
	Method (FSL=ref)	0.25 (0.07)	3.62 (61)	0.0006	(0.11, 0.39)
	Scan (Scan1=ref)	-0.14 (0.07)	-2.04 (61)	0.046	(-0.28, -0.002)
**ROI 5**	Intercept	6.36 (0.24)	26.11 (28.1)	<0.0001	(5.86, 6.86)
	Method (FSL=ref)	0.92 (0.14)	6.62 (61)	<0.0001	(0.64, 1.19)
	Scan (Scan1=ref)	-0.25 (0.14)	-1.83 (61)	0.07	(-0.53, 0.02)
ROI 6	Intercept	5.58 (0.21)	26.91 (42.2)	<0.0001	(5.16, 6.00)
	Method (FSL=ref)	-0.75 (0.17)	-4.40 (61)	*<0.0001*	(-1.09, 0.41)
	Scan (Scan1=ref)	-0.14 (0.17)	-0.84 (61)	0.40	(-0.49, 0.20)
**ROI 7**	Intercept	11.27 (0.39)	29.21 (24.9)	<0.0001	(10.47, 12.06)
	Method (FSL=ref)	1.49 (0.18)	8.39 (61)	<0.0001	(1.13, 1.84)
	Scan (Scan1=ref)	-0.19 (0.18)	-1.09 (61)	0.28	(-0.55, 0.16)

ROI 8	Intercept	10.74 (0.34)	31.62 (26.5)	<0.0001	(10.04, 11.44)
	Method (FSL=ref)	1.67 (0.18)	9.45 (61)	*<0.0001*	(1.32, 2.02)
	Scan (Scan1=ref)	-0.23 (0.18)	-1.28 (61)	0.21	(-0.58, 0.13)
**ROI 9**	Intercept	6.26 (0.23)	27.75 (27.9)	<0.0001	(5.79, 6.72)
	Method (FSL=ref)	2.00 (0.13)	15.80 (61)	*<0.0001*	(1.75, 2.26)
	Scan (Scan1=ref)	-0.11 (0.13)	-0.89 (61)	0.38	(-0.37, 0.14)
ROI 10	Intercept	10.50 (0.33)	31.72 (26)	<0.0001	(9.82, 11.18)
	Method (FSL=ref)	3.98 (0.17)	23.87 (61)	<0.0001	(3.64, 4.31)
	Scan (Scan1=ref)	-0.23 (0.17)	-1.37 (61)	0.18	(-0.56, 0.11)

β coefficient for the method *represents the difference in mean estimates between SPM and FSL when scan is fixed;* β_0 from the model equation corresponds to intercept row, β_1 corresponds to method row, β_2 corresponds to scan row.

Table 7:Intraclass correlation coefficients (ICC) (scan1, scan2) and 95% Confidence Interval (CI) for each method:

Tissue type	*ICC, FSL (Agreement) 95% CI*	*ICC, SPM (Agreement) 95% CI*
	Threshold=0	
GM	0.99 (0.976, 0.996)	0.991 (0.979,0.996)
WM	0.993 (0.982, 0.997)	0.943 (0.868,0.976)
CSF	0.945 (0.871, 0.977)	0.955 (0.893,0.981)
	**Threshold=0.5**	
GM	0.969 (0.927, 0.987)	0.994 (0.984,0.997)
WM	0.995 (0.988, 0.998)	0.985 (0.965,0.994)
CSF	0.878 (0.727, 0.948)	0.968 (0.923,0.987)
	**Threshold=0.8**	
GM	0.943 (0.866, 0.976)	0.987 (0.97,0.995)
WM	0.995 (0.987, 0.998)	0.986 (0.966,0.994)
CSF	0.889 (0.75, 0.953)	0.971 (0.93,0.988)
	**Threshold=0.95**	
GM	0.943 (0.867, 0.976)	0.959 (0.902,0.983)
WM	0.994 (0.986, 0.998)	0.982 (0.956,0.993)
CSF	0.886 (0.744, 0.952)	0.973 (0.935,0.989)

Links to code/datasets/segmentation:

1. Segmentation of one subject (scan1, scan 2)
1. (https://figshare.com/s/ab4d850eca806c6c914d,https://figshare.com/s/b2165ac278be958a64f6)
2. ROI_creation (matlab files/code): https://figshare.com/s/1b3b28858cad1c000dbe
3. ROI_volume computation: https://figshare.com/s/e3912960874d798e9421
4. ROI_volume_datafile: https://figshare.com/s/d017b21413c3d7753342

5. fslR segmentation: https://figshare.com/s/fa3479c41a1dc1e504a6
6. Tissue volume extracted data file: https://figshare.com/s/a5cd342cb3bf62ef25d5
7. R_code_descriptive stats and ICC: https://figshare.com/s/143720e6d282a597371f
8. ROI_FSL_computation: https://figshare.com/s/4949c3343051abc471cd
9. SAS_code_ROI_Analysis: https://figshare.com/s/667fb08fcf248f9b2fdd
10. SAS_codel_volume_tissue_type_analysis: https://figshare.com/s/6fd07e93d5f0627741ea
11. Rating file for plots: https://figshare.com/s/e9d12718ea42ba0000b2
12. R_code_Ratings_plots: https://figshare.com/s/d2da499312fddfd79c38
